# Molecular Characterization of an IncFII_k_ Plasmid Co-harboring *bla*_IMP–26_ and *tet*(A) Variant in a Clinical *Klebsiella pneumoniae* Isolate

**DOI:** 10.3389/fmicb.2020.01610

**Published:** 2020-07-24

**Authors:** Hong Yao, Jing Cheng, Aijuan Li, Runhao Yu, Wenbo Zhao, Shangshang Qin, Xiang-Dang Du

**Affiliations:** ^1^College of Animal Science and Veterinary Medicine, Henan Agricultural University, Zhengzhou, China; ^2^School of Pharmaceutical Sciences, Zhengzhou University, Zhengzhou, China

**Keywords:** *Klebsiella pneumoniae*, carbapenems, tigecycline, resistance, *bla*_IMP–26_, *tet*(A) variant, IncFII_k_ plasmid

## Abstract

Carbapenems and tigecycline are two important classes of antimicrobial agents to treat the infections caused by Enterobacterales. Here, we reported a plasmid carrying both *bla*_IMP–26_ and *tet*(A) variant in clinical *Klebsiella pneumoniae* KP-1572. MIC results showed that *K. pneumonia* KP-1572 was resistant to a wide range of antimicrobials. The *bla*_IMP–26_ and *tet*(A) variant were located on an identical plasmid, which was indicated by S1-PFGE and southern blotting hybridization and can be successfully transferred by electroporation. Whole-plasmid sequencing and analysis revealed that a 142,993-bp-sized plasmid, designated pIMP1572, contains an IncFII_k_ backbone and a variable region harboring *bla*_IMP–26_ and *tet*(A) variant. The plasmid pIMP1572 was apparently originated from a *tet*(A)-carrying IncFII_k_ plasmid but with a deletion length of 6,216-bp and a multiple drug resistance region (MDRR) insertion of 25,259 bp. The plasmid pIMP1572 in the present study represents the first report of the IncFII_k_ plasmid co-carrying *bla*_IMP_ and *tet*(A) variant, which should be monitored.

## Introduction

Carbapenem-resistant *Klebsiella pneumoniae* (CRKP) is an increasing problem worldwide (Nordmann et al., 2011; Munoz-Price et al., 2013). Horizontal transfer of plasmid-mediated carbapenemase-encoding genes, especially the predominant *bla*_KPC_, is contributing to the dissemination of carbapenem resistance among CRKP (Zhang et al., 2017). Unlike the *bla*_KPC_ gene, the IMP-type metallo-β-lactamase (MBL) genes, which have been reported carried by IncL/M, IncA/C, IncHI2, and IncN plasmids in Enterobacterales from Australia and China (Villa et al., 2010; Dolejska et al., 2016; Wang et al., 2017), were not frequently detected in CRKP and associated with IncFII_K_ plasmids (Wang et al., 2018). IMP-26, which differs from IMP-4 by a single amino acid substitution (Phe49Val), was firstly reported from the *Pseudomonas aeruginosa* isolate in Singapore in 2010 (Koh et al., 2010; Tada et al., 2016) and was demonstrated to possess increased carbapenem-hydrolyzing activity to meropenem than IMP-1 (Tada et al., 2016).

Tigecycline was considered to be the last-resort drug to treat infections caused by CRKP (Tasina et al., 2011). However, the previously described *tet(*A) variant (Yao et al., 2018) and recently identified *tet*(X) variants, such as *tet*(X3), *tet*(X4), *tet*(X5), and *tet*(X6) (He et al., 2019; Sun et al., 2019; Wang L. et al., 2019a; Liu et al., 2020), have been reported to mediate the low-level and high-level tigecycline resistance, respectively. Both *tet*(A) variant and *tet*(X) variants are mobilized, indicating that they are posing a higher threat to public health. The association between the tigecycline resistance genes and carbapenem-hydrolyzing enzymes genes in CRKP has not been well explored.

Herein, we characterized an IncFII_k_ plasmid co-carrying *bla*_IMP–26_ and tigecycline-resistant gene *tet*(A) variant in a clinical *K. pneumoniae* isolate which displayed resistance to carbapenems and tigecycline.

## Materials and Methods

### Bacterial Isolation, Antimicrobial Susceptibility Testing, and PCR Detection

*Klebsiella pneumonia* KP-1572 was obtained from a sputum culture of a 1-day newborn boy hospitalized due to intracranial hemorrhage associated with neonatal infections at a teaching hospital of the Zhengzhou University.

The MICs to imipenem, meropenem, aztreonam, ceftazidime, gentamicin, amikacin, tetracycline, tigecycline, colistin, and fosfomycin were determined using the broth microdilution method and the agar dilution method (for fosfomycin) according to the Clinical and Laboratory Standards Institute guidelines (CLSI) (CLSI, 2020) and the European Committee on Antimicrobial Susceptibility Testing (EUCAST)^1^. *Escherichia coli* ATCC 25922 served as the quality-control strain.

PCR was used to determine the presence of the carbapenem-resistance and tigecycline-resistance genes *bla*_KPC_, *bla*_NDM_, *bla*_IMP_, *bla*_VIM_, *bla*_OXA,_ *tet*(A), and *tet*(X4) with primers described previously (Poirel et al., 2011; Yao et al., 2018; He et al., 2019).

### Multilocus Sequence Typing

Multilocus sequence typing (MLST) of *K. pneumoniae* KP-1572 were performed as described previously (Diancourt et al., 2005). PCR amplification and sequencing for seven housekeeping genes (*gapA*, *infB*, *mdh*, *pgi*, *phoE*, *rpoB*, and *tonB*) were carried out. Then, the sequences of these seven housekeeping genes were submitted to a database^2^ to obtain the ST type.

### S1-PFGE and Southern Blotting

S1-PFGE and Southern blotting were performed to detect the location of the resistance genes. The whole-cell DNA of the *K. pneumonia* KP-1572 isolate in agarose gel plug was treated with S1 nuclease (TaKaRa, Dalian, China) and then separated by PFGE under the conditions reported previously (Qin et al., 2014). The location of the *bla*_IMP–26_ and *tet*(A) variant was indicated by Southern hybridization using a digoxigenin-labeled *bla*_IMP_ and *tet*(A) probe, respectively, according to the manufacturer’s instructions for the DIG-High Prime DNA Labeling and Detection Starter Kit II (Roche Diagnostics, Basel, Switzerland).

### Conjugation Assay and Electrotransformation Experiments

Conjugation assays were performed according to the method described previously with minor modification (Borgia et al., 2012). Briefly, the rifampicin-resistant *E. coli* isolate EC600 was used as the recipient, and donor and recipient strains were mixed at a ratio of 1:4 on LB agar and cultured for 12 h. The mixtures were collected and then plated on an LB agar containing rifampicin (64 μg/mL) and meropenem (1 μg/mL) or tigecycline (0.5 μg/mL). Electrotransformation was performed as described previously (Yan et al., 2020). Briefly, the plasmid co-harboring the *bla*_IMP–26_ and *tet*(A) variant was extracted from *K. pneumoniae* KP-1572 and then transferred into the recipient Electro-Cells *E. coli* DH5α (TaKaRa, Dalian, China) by electroporation (Bio-Rad MicroPulser, 1.8 kV, 5 ms). The electrotransformants were screened by LB agar containing meropenem (1 μg/mL).

### Plasmid Sequencing and Analysis

The plasmid was sequenced by the PacBio RS and Illumina MiSeq platforms (Shanghai Personal Biotechnology Co., Ltd., China). The PacBio sequence reads were assembled with HGAP4 and CANU (Version 1.6) and corrected by Illumina MiSeq with Pilon (Version 1.22). The prediction and annotation of ORFs were performed using Glimmer 3.0.

## Results and Discussion

*Klebsiella pneumoniae* KP-1572 exhibited a multiple drug resistance (MDR) profile for a wide range of antimicrobial agents, including imipenem, meropenem, aztreonam, ceftazidime, gentamicin, tetracycline, tigecycline, and colistin, while it was susceptible to amikacin and fosfomycin (Table 1). Resistance gene screening and sequencing revealed that *K. pneumonia* KP-1572 co-carried the carbapenem-resistance gene *bla*_IMP–26_ variant and tigecycline-resistance gene *tet*(A) variant. The *tet*(A) variant showed a mutation profile of I5R, V55M, I75V, T84A, S201A, F202S, and V203F compared with *tet*(A) (X00006) (Waters et al., 1983) and exhibited 100% identity with that in our previous study (Yao et al., 2018). Multilocus sequence typing (MLST) showed that *K. pneumonia* KP-1572 belonged to uncommon sequence type ST1083, which was reported in carbapenem*-*resistant *K. pneumonia* isolated from clinical bovine mastitis in Tunisia (Saidani et al., 2018).

**TABLE 1:** Antibiotic susceptibility of KP-1572 isolate and its electrotransformant.

Isolate	Antibiotic susceptibility (μg/ml) to									
	IPM^a^	MEM	ATM	CAZ	GN	AK	TET	TIG	CL	FOS
KP-1572	64	>64	64	>64	64	8	>64	2	8	8
DKP1572	16	16	8	64	32	1	>64	2	0.5	<1
DH5α	<0.25	<0.25	<0.25	0.5	0.25	0.5	<0.25	<0.25	<0.25	<1

*^*a*^IPM, imipenem; MEM, meropenem; ATM, aztreonam; CAZ, ceftazidime; GN, gentamicin; AK, amikacin; TET, tetracycline; TIG, tigecycline; CL, colistin; FOS, fosfomycin.*

S1 nuclease PFGE and Southern blotting confirmed that the gene *bla*_IMP–26_ and *tet*(A) variant were located on an identical plasmid of KP-1572 (Supplementary Figure S1). The conjugation experiments failed after three attempts; however, transformants were successfully obtained by electroporation, which was confirmed by PCR and S1-PFGE (Supplementary Figure S1). The susceptibility testing results indicated that the electrotransformant (designed DKP1572) showed > 64-fold increased resistance to meropenem and imipenem compared to the recipient DH5α. DKP1572 also exhibited an increased resistance level (2 μg/mL, eightfold increase) to tigecycline than that of DH5α (Table 1).

**FIGURE 1:**
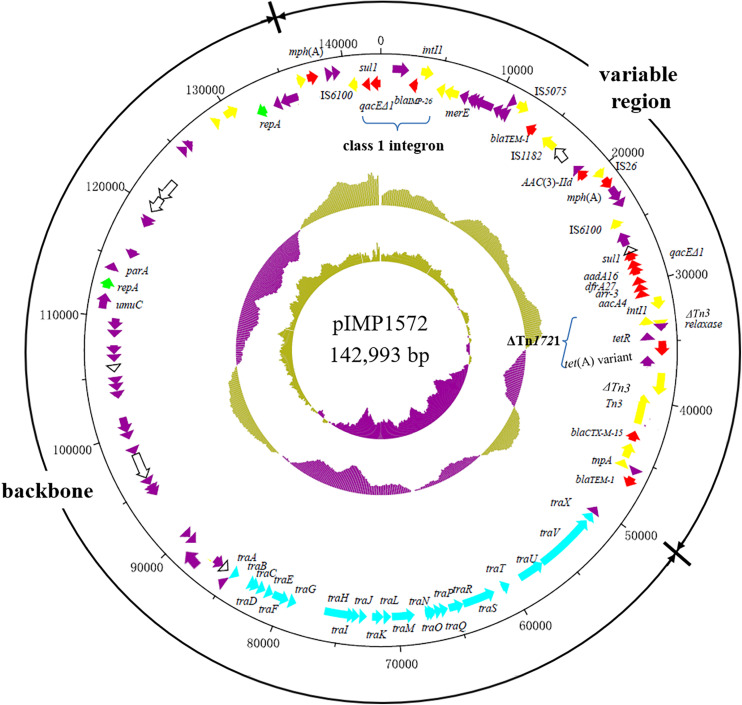
The structure of the plasmid pIMP1572 from *K. pneumonia* KP-1572. The size scale in bp; genes are color-coded, depending on functional annotations: red, antimicrobial resistance; blue, plasmid transfer; green, plasmid replication; yellow, transposition; purple, other functions; and white, hypothetical proteins.

Whole-plasmid sequencing of plasmid in DKP1572 (named pIMP1572) showed that it is an IncFII_k_-type plasmid with a length of 142,993 bp and an average GC content of 53.5%, which encodes 117 predicted open reading frames. The plasmid pIMP1572 consisted of an 89,521-bp IncFII_K_ typical backbone encoding genes responsible for plasmid replication, transfer, and stability functions, and a 53,472-bp variable region (Figure 1). The oriTs, relaxases, T4SS gene clusters, and T4CPs are closely associated with conjugation of plasmids (Li et al., 2018); however, mutations were present in relaxases, TraB, TraD, and T4CP encoding genes in pIMP1572, which might explain the failure of its conjugation. pIMP1572 is a multiple-drug-resistance plasmid that included the aminoglycoside resistance genes *aac*(3)-IId, *aadA16*, and *aac*(6′)*-Ib-cr*; β-lactam resistance genes *bla*_TEM–1B_, *bla*_CTX–M–15_, and *bla*_TEM–1C_; macrolide resistance gene *mph*(A); rifampicin resistance gene *arr-3*, sulfonamide resistance gene *sul1*; and trimethoprim resistance gene *dfrA27* in addition to the *bla*_IMP–26_ and *tet*(A) variant (Figure 1). Multiple transfer elements, such as IS*26*, were also present in this plasmid (Figure 1), which may promote the dissemination of resistance genes among *K. pneumoniae* and other Enterobacterales.

Analysis of the flanking regions of *bla*_IMP–26_ revealed that this gene was located in a class 1 integron cassette, *IntI1*-*bla*_IMP–26_-ORF1-*qacE*△*1-sul1*, which contained a complete 5′ conserved sequence (5′-CS, integrase *intl1*) and 3′-CS (*qacE*△*1-sul1*). The *bla*_IMP–26_-carrying class 1 integron cassette in this study showed 100% identity and 97% query coverage with the corresponding region of a plasmid pIMP26 in *Enterobacter cloacae* isolated from the bloodstream in China (Wang S. et al., 2019b) but was different from that reported in *P. aeruginosa* in Vietnam (Tada et al., 2016). Tn*1721* was a member of Tn*3*-family unit transposons, with the complete genetic structure of *mcp*-*res*-*tnpR*-*tnpA*-*tetR*-*tet*(A)-*pecM*-Δ*tnpA* (Allmeier et al., 1992). In this study, the *tet*(A) variant was found in a truncated Tn*1721*-like transposon with arrangement of the Δ*tnpA*-relaxase-*tetR*-*tet*(A) variant (Figure 1). Recently, the *tet*(A) variant was found located on a *bla*_KPC–2_-carrying plasmid in *K. pneumonia* and was confirmed to mediate tigecycline resistance (Yao et al., 2018; Zeng et al., 2020). To our knowledge, the current study is the first time to report the presence of a *bla*_IMP–26_ and *tet*(A) variant-co-carrying plasmid, which can render *K. pneumonia* to be reduced susceptibility significantly to both carbapenems and tigecycline, posing a threat to treatments of CRKP infection in clinic.

**FIGURE 2:**
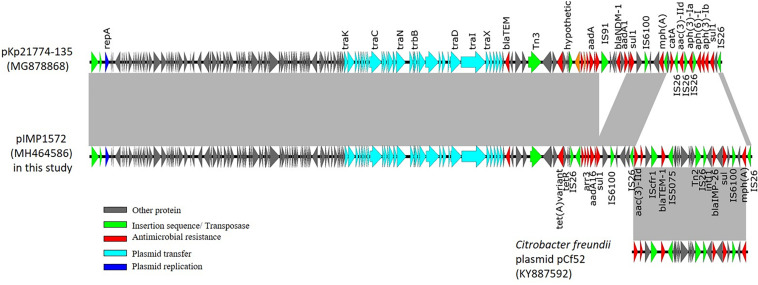
The structure comparison of the plasmid pIMP1572 identified in this study with others published previously. The gray-shaded areas represent genomic regions that share 99% nucleotide sequence identities.

The sequence data revealed that pIMP1572 shares 99.99% identity and 89% query coverage^3^ with an IncFII_k_ type pKp21774-135 in *K. pneumoniae* (accession number in GenBank, MG878868) (Figure 2). Multiple drug resistance regions (MDRR) with a length of 25,259 bp insertion and 6,216 bp deletion were found in pIMP1572 plasmids in this study, when compared with pKp21774-135 (Figure 2). The insertion MDRR that contained multiple resistance genes was bracketed by IS*26*, including *qacE*△*1-sul1*, *bla*_TEM–1_, *aac*(3)-IId, and *mph*(A) in addition to *bla*_IMP–26_. The sequence of the MDRR region showed 99% identity and query coverage to the corresponding region of an IncA/C2 plasmid pCf52 (KY887592) from *Citrobacter freundii* (Figure 2), indicating that this MDRR may be acquired from *C. freundii* other than *K. pneumonia.*

IncFII_k_ plasmid, a member of the divergent IncFII replicon plasmids, played a significant role in restoring and transferring the *bla*_KPC_ gene in *K. pneumoniae* (Feng et al., 2017; Wang et al., 2017; Bi et al., 2018; Fu et al., 2019), which was also reported sporadically to carry MBL-encoding genes, such as *bla*_NDM_ (Sugawara et al., 2019). The plasmid pIMP1572 identified in this study is different from previously reported *bla*_IMP_-harboring plasmids that belonged to incompatibility groups IncL/M, A/C, HI2, and IncN (Carattoli, 2009; Dolejska et al., 2016; Wang et al., 2017) and represents the first report of IncFII_k_ plasmid carrying *bla*_IMP_. Association of *bla*_IMP_-like genes with an epidemic IncFII_k_ plasmid may facilitate their further dissemination among *K. pneumonia*. Thus, enhanced efforts should be made to monitor the potentially rapid dissemination of *bla*_IMP_ and *tet*(A) variant-encoding IncFII_k_-type plasmid.

## Data Availability Statement

The datasets presented in this study can be found in online repositories. The names of the repository/repositories and accession number(s) can be found at: https://www.ncbi.nlm.nih.gov/genbank/, MH464586.

## Ethics Statement

The studies involving human participants were reviewed and approved by the Ethics Review Committee of Life Sciences of Zhengzhou University. Written informed consent to participate in this study was provided by the participants’ legal guardian/next of kin. Written informed consent was obtained from the individual(s), and minor(s)’ legal guardian/next of kin, for the publication of any potentially identifiable images or data included in this article.

## Author Contributions

SQ and X-DD designed the research and supervised the study. HY, JC, RY, AL, and WZ performed the experiments and analyzed the data. HY, JC, and X-DD wrote the manuscript. All authors revised the manuscript and approved the final version for submission.

## Conflict of Interest

The authors declare that the research was conducted in the absence of any commercial or financial relationships that could be construed as a potential conflict of interest.

## Footnotes

1 http://www.eucast.org

2 https://bigsdb.pasteur.fr/cgi-bin/bigsdb/bigsdb.pl?db=pubmlst_klebsiella_isolates

3 https://blast.ncbi.nlm.nih.gov/

## Supplementary Material

FIGURE S1 Detection of *bla*_IMP26–_ and *tet*(A) variant- co-carrying plasmid by S1-PFGE and Southern hybridization.
