# Supplementary figures and images for: Molecular Characterization of an IncFIIk Plasmid Co-harboring blaIMP–26 and tet(A) Variant in a Clinical Klebsiella pneumoniae Isolate
Source: Front Microbiol. 2020 Jul 24;11:1610. doi: 10.3389/fmicb.2020.01610 (PMC7393768; doi:10.3389/fmicb.2020.01610)

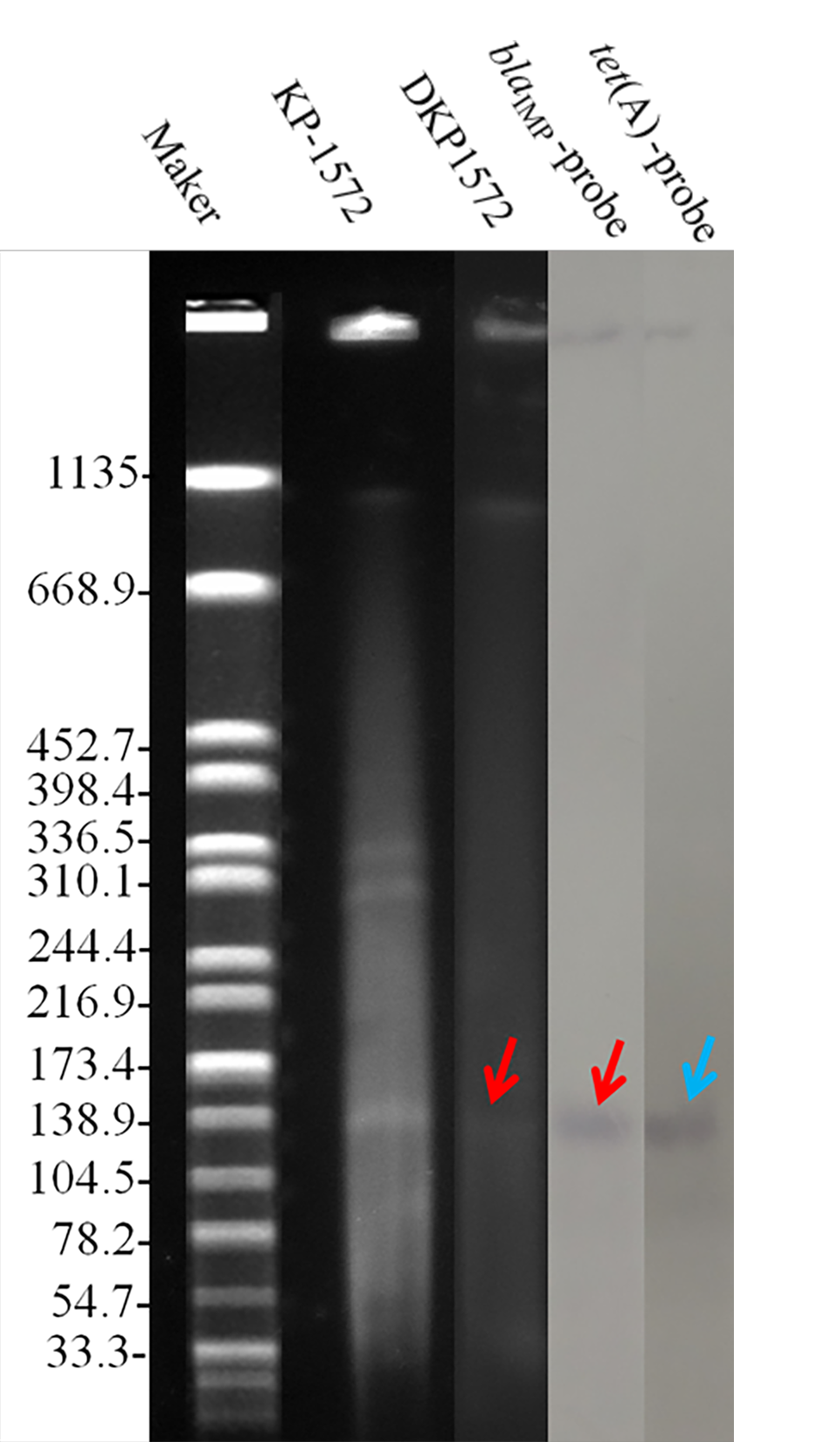

Supplement: FIGURE S1 — Detection of blaIMP26– and tet(A) variant- co-carrying plasmid by S1-PFGE and Southern hybridization. [file Image_1.TIF]
